# Supplementary material for: Cut-Out Towne-View Whole-Brain 320-Row Four-Dimensional Computed Tomography Angiography for Assessing the Anterior Intracranial Collateral Status: A Retrospective Study
Source: Diagnostics (Basel). 2022 May 27;12(6):1336. doi: 10.3390/diagnostics12061336 (PMC9221849; doi:10.3390/diagnostics12061336)
Supplement: Supplementary file 1 [file diagnostics-12-01336-s001.zip › Figures S1.and S2.pdf]

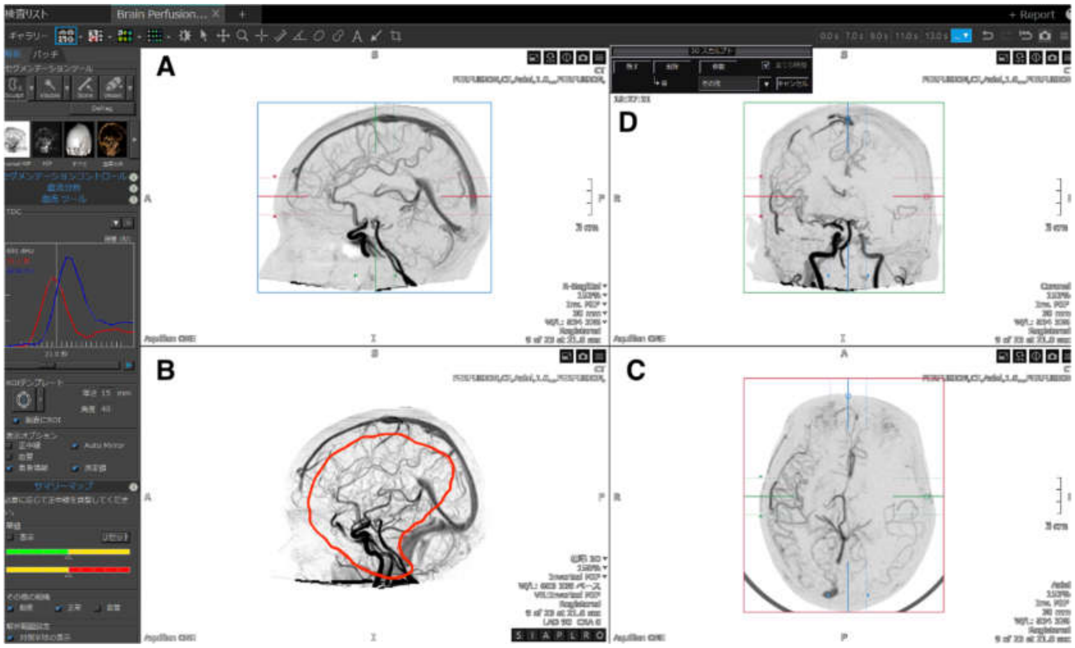

Figure S1. A screenshot of cutting out intracranial arteries (B: red line) in the Vitrea workstation.

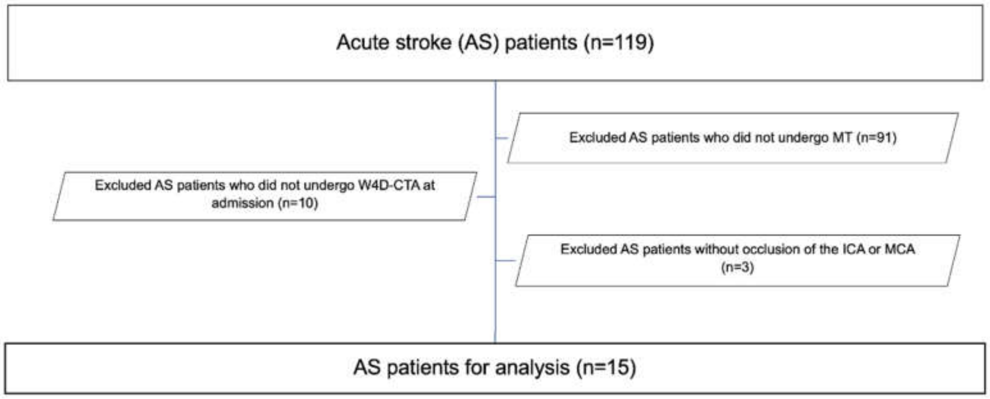

Figure S2. Flow chart of patient selection for the analysis. ICA, internal carotid artery; MCA, middle cerebral artery; MT, mecanocal thrombectomy; W4D-CTA, whole-brain four-dimensional computed tomography angiography.
