# Supplementary material for: Cut-Out Towne-View Whole-Brain 320-Row Four-Dimensional Computed Tomography Angiography for Assessing the Anterior Intracranial Collateral Status: A Retrospective Study
Source: Diagnostics (Basel). 2022 May 27;12(6):1336. doi: 10.3390/diagnostics12061336 (PMC9221849; doi:10.3390/diagnostics12061336)
Supplement: Supplementary file 1 [file diagnostics-12-01336-s001.zip › Table_S1_Dose.pdf]

Table S1 Number of volume scanning and radiation dose.

Case	Number of VS	Total CTDI vol (mGy)	Total DLP (mGy·cm)
1	25	96.6	1545
2	26	100.46	1606.8
3	23	88.91	1421.4
4	25	96.6	1545
5	27	104.32	1668.6
6	24	92.73	1483.2
7	24	92.73	1483.2
8	24	92.73	1483.2
9	26	100.46	1606.8
10	18	69.56	1112.4
11	26	100.46	1606.8
12	27	104.32	1668.6
13	30	115.91	1854
14	23	88.91	1421.4
15	25	96.6	1545
Median	25	96.6	1545

CTDI vol, computed tomography dose index volume; DLP, dose-length product; VS, volume scanning.
