# Supplementary material for: Cut-Out Towne-View Whole-Brain 320-Row Four-Dimensional Computed Tomography Angiography for Assessing the Anterior Intracranial Collateral Status: A Retrospective Study
Source: Diagnostics (Basel). 2022 May 27;12(6):1336. doi: 10.3390/diagnostics12061336 (PMC9221849; doi:10.3390/diagnostics12061336)
Supplement: Supplementary file 1 [file diagnostics-12-01336-s001.zip › Table_S2_Collateral_evaluation.pdf]

Table 2. Evaluation of anterior intracranial collateral status based on unedited AP-view and cut-out Towne-view 4D-CTA images.

Case	Occlusion	Not edited AP-view 4D-CTA Raters				Cut-out Towne-view 4D-CTA Raters			
		A	B	C	D	A	B	C	D
1	MCA	P	I	I	I	I	P	I	I
2	MCA	P	I	I	I	I	G	I	I
3	ICA	P	P	P	G	P	P	P	P
4	MCA	I	I	I	G	I	I	I	I
5	MCA	P	G	P	G	G	G	G	G
6	MCA	I	I	I	I	I	I	I	P
7	ICA	I	I	I	G	G	G	G	G
8	ICA	G	G	G	I	G	G	G	G
9	MCA	P	P	P	I	P	I	I	P
10	MCA	P	G	P	I	I	I	I	I
11	ICA	G	G	G	P	G	G	G	G
12	MCA	G	I	I	I	I	I	I	I
13	ICA	I	I	I	P	I	I	I	I
14	MCA	I	P	P	P	I	P	P	I
15	MCA	I	I	I	I	I	I	I	I

4D-CTA, four-dimensional computed tomography angiography; AP, anteroposterior; G, good; I, intermediate; ICA, internal carotid artery, MCA, middle cerebral artery; P, poor.
